# Ocean currents break up a tabular iceberg

**DOI:** 10.1126/sciadv.abq6974

**Published:** 2022-10-19

**Authors:** Alex Huth, Alistair Adcroft, Olga Sergienko, Nuzhat Khan

**Affiliations:** ^1^ AOS Program, Princeton University, Princeton, NJ 08540, USA.; ^2^ Macaulay Honors College at Hunter College, City University of New York, New York, NY 10023, USA.

## Abstract

In December 2020, giant tabular iceberg A68a (surface area 3900 km^2^) broke up in open ocean much deeper than its keel, indicating that the breakage was not immediately caused by collision with the seafloor. Giant icebergs with lengths exceeding 18.5 km account for most of the calved ice mass from the Antarctic Ice Sheet. Upon calving, they drift away and transport freshwater into the Southern Ocean, modifying ocean circulation, disrupting sea ice and the marine biosphere, and potentially triggering changes in climate. Here, we demonstrate that the A68a breakup event may have been triggered by ocean-current shear, a new breakup mechanism not previously reported. We also introduce methods to represent giant icebergs within climate models that currently do not have any representation of them. These methods open opportunities to explore the interactions between icebergs and other components of the climate system and will improve the fidelity of global climate simulations.

## Teaser

A mechanism of iceberg breakup is revealed by simulations.

## INTRODUCTION

The Antarctic Ice Sheet loses its mass to the Southern Ocean via two mechanisms—sub–ice-shelf melting and iceberg calving (*1*, *2*). Giant tabular icebergs with areas exceeding 100 km^2^ comprise 89% of the total volume of all Antarctic icebergs (*3*), and the largest iceberg area on record exceeded 10,000 km^2^ (*4*). After calving, icebergs drift into the open ocean where they influence large-scale ocean circulation by depositing cold and fresh meltwater to the ocean surface, which modifies the vertical ocean density profile and affects deep water formation (*5*–*7*). Geologic evidence implicates icebergs in abrupt changes of the climate system, including the modulation of glacial-interglacial cycles by large Antarctic icebergs (*8*) and Heinrich events (*9*, *10*).

Despite their importance to the climate system, large tabular icebergs are not represented in current climate models, which typically only account for icebergs with areas smaller than ∼3.5 km^2^. There are a number of challenges that explain why climate models do not represent giant tabular icebergs, including the practical assumption that the modeled icebergs are smaller than the ocean model cell size (*11*) and a need to better account for the processes of iceberg decay (*12*, *13*). Omission of the largest icebergs results in an inaccurate modeled distribution of iceberg meltwater because iceberg size influences drift trajectories and decay rates. For instance, small icebergs melt quicker than large icebergs (*14*, *15*). The impact of icebergs on climate cannot be assessed without an accurate representation of their drift trajectories, breakup, and meltwater distribution.

To address this issue, we have developed a bonded-particle iceberg model (see Materials and Methods) to be a component of a coupled climate model. This modeling framework—the improved Kinematic Iceberg Dynamics (iKID)—represents a giant tabular iceberg as a collection of bonded elements that evolve in response to atmospheric and oceanic forcings. The bonds between the elements can break, mimicking breakup of the iceberg. In this study, we use the iKID framework to investigate evolution of the iceberg A68a.

**Fig. 1.**
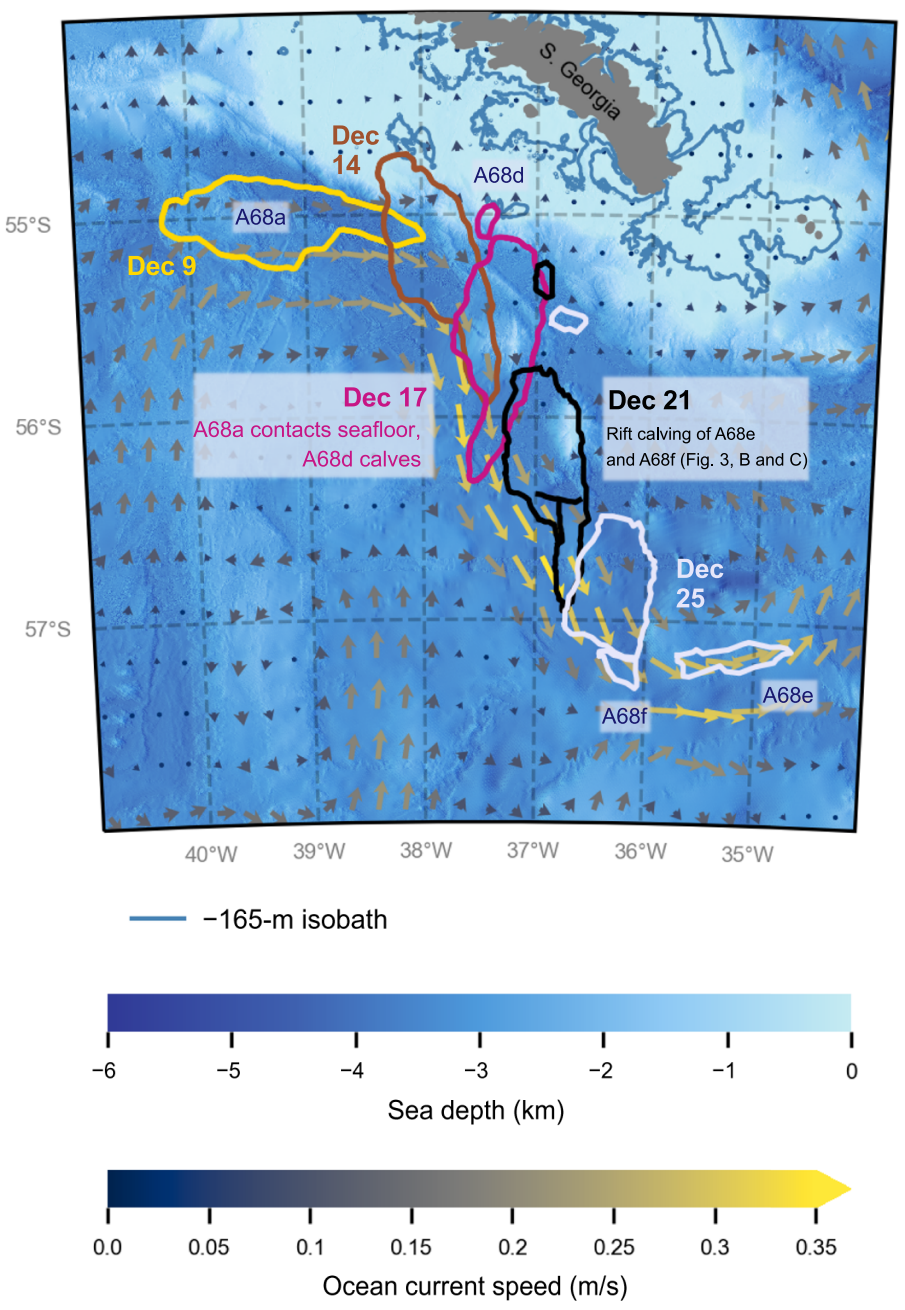
The two observed rift calving breakup events of iceberg A68a in December 2020. Outlines of the observed A68a and its child icebergs (derived from NASA MODIS and ESA Sentinel-1) are plotted over the sea depth (*34*, *35*) and the December 16 ocean current velocities (*22*). The −165-m isobath approximates the sea depth at which the keel of A68a would contact the seafloor.

Iceberg A68 (surface area 5800 km^2^) calved from the Larsen C Ice Shelf (Antarctic Peninsula) between the 10 and 12 July 2017 and, a few days later, split into two bergs, the 5710 km^2^ A68a and 90 km^2^ A68b (*16*, *17*). By December 2020, iceberg A68a had reduced in area to 3900 km^2^ while drifting north to near South Georgia Island. Here, A68a experienced two rift calving events (Fig. 1), whereby large (>5 km^2^) child bergs break off from a larger parent berg (*18*). The first rift calving event occurred on December 17 near a shallow topographic feature and was likely triggered when A68a briefly contacted the seafloor (*16*). However, the cause of the second rift calving event, where the “finger” that comprised the southern half of A68a broke off around December 21, was unclear. Because it occurred in deep water, it was not immediately triggered by local contact with the seafloor.

We hypothesize that a lateral gradient in ocean current along the iceberg may have induced horizontal iceberg stresses that caused the rift calving event of December 21. To test this idea, we simulate the evolution of iceberg A68a from December 9 to 23 using the iKID model, forced by ocean currents, sea surface slopes, and wind stress derived from satellite datasets (see the “Experimental setup” section). Our goal is to capture the observed drift and both breakup events with a single set of realistic model parameters. Using this event as a test case, we aim to demonstrate that the iKID model is accurate and computationally efficient enough to couple with climate models.

## RESULTS

**Fig. 2.**
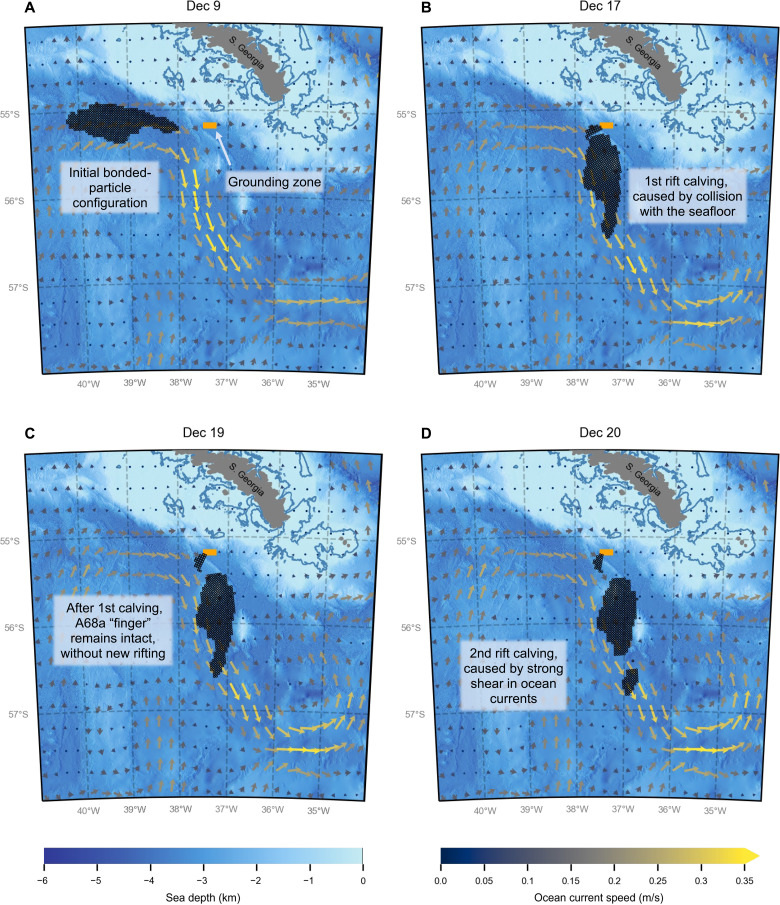
The simulated drift and decay of iceberg A68a in December 2020. (**A**) The initial position of the bonded-particle iceberg on December 9. (**B**) The first rift calving event upon contacting the seafloor (December 17). (**C**) The iceberg configuration on December 19, where the finger is still intact. (**D**) The rift calving of the iceberg finger caused by strong shear in ocean currents (December 20). The icebergs are plotted over the sea depth (*34*, *35*) and ocean current velocities (*22*).

**Fig. 3.**
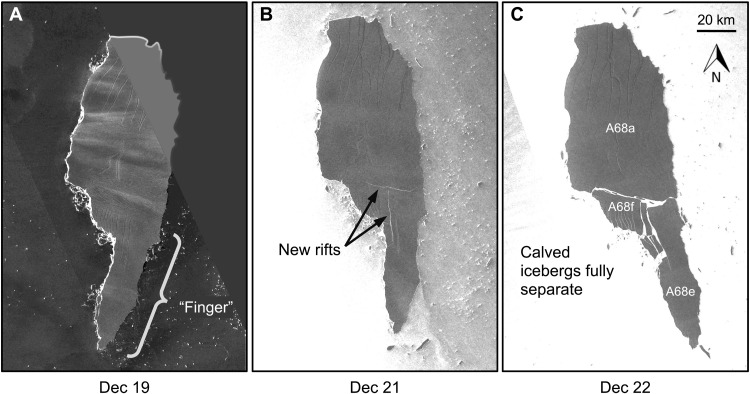
Sentinel-1 imagery of iceberg A68a. (**A**) December 19 (partial image). (**B**) December 21. (**C**) December 22. The full separation of the calved finger is apparent on December 22 (C) but was preceded by new rifting on December 21 (B) that was previously absent on December 19 (A).

The simulated iceberg trajectory, orientation, and fracture (Fig. 2 and movie S1) reasonably match observations (Fig. 1). Over the first week of the simulation, the iceberg rotated ∼90° clockwise and collided with the grounding zone around December 17, causing iceberg A68d to calve from its northern tip (Fig. 2B). Afterward, the remainder of the modeled A68a drifted southeast. In agreement with satellite observations (Fig. 3A), the modeled finger remained fully intact through December 19 (Fig. 2C). However, around December 20 (Fig. 2D), the finger of the modeled A68a became positioned within a stronger, more eastward ocean current than the rest of the iceberg. The resulting lateral tension on the iceberg, induced by this strong shear in ocean currents, caused the finger to calve off. Satellite imagery shows that the observed iceberg was similarly positioned within the ocean currents during this second calving event (Fig. 1, black outline), where the finger was at least partially separated by December 21 (Fig. 3B) and fully detached by December 22 (Fig. 3C).

## DISCUSSION

Our simulation demonstrates that the shear of ocean currents may be a cause of some iceberg break ups, which has not been reported previously for the evolution of icebergs in the open ocean. This conclusion is supported by the fact that both the collision-induced and open-ocean breakup events were captured using the same realistic tensile ice strength (see Materials and Methods). It is likely that the shape of an iceberg dictates its susceptibility to rift calving, as longer icebergs such as A68a are more prone to the stresses that a strong gradient in ocean current exerts on their body. Preexisting cracks in an iceberg probably increase susceptibility to current-induced rift calving as well. Rift calving can preferentially occur along these preexisting cracks, which typically develop on an iceberg before it calves from an ice shelf (*19*, *18*). While we do not incorporate preexisting cracks into the model here, potential schemes to account for these cracks include lowering the ice strength on certain bonds or adding a damage model, where some bonds are initialized with preexisting damage.

We further conclude that the iKID module represents a substantial advance over the simpler point-particle iceberg modules that are typically coupled with climate models (*5*–*7*). By accounting for external forces that vary across an iceberg body, the bonded-particle model not only allows for accurate representation of rift calving but also improves representation of drift by capturing features such as the rotation of A68a.

The identification of ocean-current shear as a potential iceberg-breakup mechanism using a numerical model suggests that such models have practical applications beyond simulation of a single event. These modeling tools may be used along with other existing tools such as remote sensing and in situ (when available) observations to investigate evolution of icebergs and their interactions with the ocean, atmosphere, sea ice, and biosphere. Our simulations also illustrate that these kinds of models can be computationally efficient (see Materials and Methods) to be used as a component of climate models yet still accurate enough to simulate observed drift and fracture. These modeling capabilities are a breakthrough that will allow investigations into interactions between icebergs and other components of the climate system and to improve the fidelity of climate models.

## MATERIALS AND METHODS

The iKID model treats each particle, or element, as a vertical column of ice that experiences drag from the ocean, atmosphere, sea ice, and seafloor (if grounded); normal forces, shear, and torques between elements (*20*); a force due to sea surface slope; a wave radiation force; and the Coriolis force. When the tensile stress on a bond exceeds the tensile ice strength, the bond breaks (*21*). While each element has a horizontal area and thickness, the iKID model is two-dimensional in the sense that there is only one vertical layer of elements and only horizontal forces are represented. Therefore, bending effects associated with grounding or changes in dynamic ocean topography over the length of the iceberg are neglected.

### Computational efficiency

To increase computational efficiency, we developed a multiple time step (MTS) velocity Verlet scheme to integrate the equations of motion. In the MTS scheme, all forces are evaluated on a “long” time step increment (here, 30 min), except for the grounding drag and interactive forces between elements belonging to the same “conglomerate” of bonded elements, which are evaluated more frequently over a series of shorter substeps small enough to guarantee stability (here, 20 s). This scheme reduces how often each force must be evaluated, decreases the number of interpolations of gridded data to particles, and minimizes memory transfers between processing domains during parallel runs.

When the A68a experiment was run in serial on an Intel Xeon CPU ES-2697 v4, the wall clock time for the MTS scheme averaged about 10 s to simulate 1 day of iceberg evolution. Parallelized runs achieve similar wall clock times because all bonded particles comprising an iceberg conglomerate are transferred to all processing domains that the conglomerate overlaps (see Supplementary text) and computed redundantly. Therefore, the conglomerate with the greatest number of particles has a strong influence over how quickly a simulation will run. Nevertheless, iceberg A68a was the sixth largest iceberg on record (*4*), so we conclude that our bonded-particle model is computationally efficient enough for implementation within century-scale climate simulations. Further speed up may be possible through vectorization or by increasing the “short” MTS time step increments, which may be possible without sacrificing stability if particle size is increased or the Young’s modulus is decreased (see the “Tuning” section).

### Experimental setup

We forced the A68a simulation with OSCAR (Ocean Surface Current Analysis Real-time) near-surface ocean current velocities (*22*), SSALTO/DUACS (Segment Sol multimissions d’ALTimétrie, d’Orbitographie et de localisation précise/Data Unification Altimeter Combination System) sea surface heights (*23*), and NCEP/NCAR (National Centers for Environmental Prediction/National Center for Atmospheric Research) Reanalysis 1 10m vector winds (*24*). These fields were interpolated to the particles from a 1/8° background grid at the start of each half-hour time step. Guided by NASA Aqua MODIS (Moderate Resolution Imaging Spectroradiometer) imagery, we initialized the iceberg position within the OSCAR current at its observed longitude on 9 December 2020, and we assigned the iceberg an initial eastward velocity of 0.22 m/s. We arranged the bonded particles on a regular Cartesian lattice, i.e., square packing, where each particle has a maximum of four bonds, a constant radius of 1.5 km, and an estimated ice thickness of 200 m. Figure 2A shows this initial December 9 iceberg configuration, where the orange rectangle marks the grounding zone responsible for the first breakup event and is the only area where we activate the iceberg grounding drag. We manually delineated this zone to be slightly southwest of the observed grounding zone because the OSCAR ocean currents do not appear to flow close enough to the observed grounding zone.

### Tuning

The primary tuning parameters that affect model behavior are the Young’s modulus (*E*), the horizontal (*c*_o, h_) and vertical (*c*_v, h_) ocean drag coefficients, the grounding drag coefficient (*c*_g_), and the tensile bond strength (σ_c_). A full description of these parameters is provided in the Supplementary text, and the values of all model parameters used for the A68a experiment are given in table S1. We caution that these values may not be applicable for all icebergs. Therefore, additional icebergs should be modeled in future studies to better constrain these values and estimate how they may vary between icebergs. We describe our tuning process here as a guide for future studies.

We began the tuning process by determining an appropriate Young’s modulus, *E*. Pure, undamaged ice has a Young’s modulus between 1 and 10 GPa. However, there are both numerical and physical reasons to decrease *E* when modeling icebergs. Numerically, smaller values of *E* decrease the velocity of seismic waves, which increases computational efficiency by allowing longer time steps when evaluating bonded-particle interactive forces. Physically, *E* should decrease under the following conditions that may be applicable to icebergs: (i) when ice temperature increases and under strain rate effects over long loading times (*25*); (ii) when seawater or surface meltwater infiltrates into firn (*26*, *27*); and (iii) to account for crevassing, which decreases the ice thickness along which stresses are transmitted (*28*). Iceberg A68a exhibited substantial crevassing that was present when it was part of the Larsen C ice shelf (*29*, *30*). We set *E* to 5 MPa, which is a large enough value to guarantee that iceberg behavior is visually stiff while allowing the bonded-particle scheme to be computationally efficient enough to use within climate models.

Next, we determined the ocean drag coefficients that yielded a modeled iceberg drift path that best matched observations: *c*_o, h_ = 0.02136 and *c*_o, v_ = 16.02. These coefficients differ from those typically used for unbonded, point-particle models of small icebergs (*6*, *31*), because here, each particle only constitutes a portion of a large bonded-particle conglomerate. Furthermore, the tuning may make up for error in the ocean current data or the fact that we force our model with ocean surface currents alone rather than currents that vary over the depth of the iceberg. When determining the optimal coefficients, we assumed for simplicity that the coefficients retained the 1:750 ratio for *c*_o, h_:*c*_o, v_ that is used in point-particle iceberg models (*6*, *31*). Other values may yield a similar model response. Note that we do not pursue a similar tuning exercise for the wind drag, because wind contribution to the motion of large icebergs is small (*32*). Instead, we simply use the wind drag coefficients from a point-particle iceberg model (*6*). We do not tune the sea-ice drag coefficients because sea ice is absent in the vicinity of the iceberg in December 2020. We are able to attribute each breakup event to contact with either the seafloor or ocean currents because these are the primary processes that determine iceberg stresses and drift.

After finalizing the Young’s modulus and ocean drag coefficients, we tuned the grounding coefficient, *c*_g_. Small values of *c*_g_ will only slow the iceberg drift, while larger values resemble an impact event, which is more appropriate for A68a. We set *c*_g_ = 10^4^ kg m^−2^ s^−1^. Last, we determined the tensile ice strength, or tensile stress threshold for breaking bonds (σ_c_), that gives the best match between modeled and observed iceberg breakup. We set σ_c_ to 18 kPa, which is similar to the stress scale of order 10 kPa estimated in a previous study for rift calving of large tabular icebergs (*33*). This previous estimate neglected local stress amplification near rift tips, an assumption that is also likely applicable to the current study given the coarse resolution of the model, where each bond is ∼3 km long.

The above tuning exercise may not have arrived at the only combination of parameters, e.g., tuning of Young’s modulus and ocean drag coefficients, sufficient to model A68a, nor did we document the sensitivity to parameters, which could be nonlinear in response and thus make particular tuning nonunique or not robust. For example, it has yet to be shown that the tuning for an iceberg would be optimal as ice thickness changes over time. Note that tuning is not a trivial process; small changes to a single parameter can sometimes divert the drift trajectory of an iceberg drastically due to strong spatial variations in ocean and climate forcings. Despite tuning sensitivity, the existence of at least one set of parameters that appear to explain the A68a breakup suggests that we may be able to find parameters for other icebergs and events, and ultimately, one can imagine a general model for these parameters.

## Supplementary Materials

### This PDF file includes:

Supplementary Text Table S1 References
