# Supplementary material for: Ocean currents break up a tabular iceberg
Source: Sci Adv. 2022 Oct 19;8(42):eabq6974. doi: 10.1126/sciadv.abq6974 (PMC9581483; doi:10.1126/sciadv.abq6974)
Supplement: Supplementary file 1 — Supplementary Text Table S1 References [file sciadv.abq6974_sm.pdf]

Science Advances
AAAS

# Supplementary Materials for

## Ocean currents break up a tabular iceberg

Alex Huth *et al.*

Corresponding author: Alex Huth, ahuth@princeton.edu

*Sci. Adv.* **8**, eabq6974 (2022)
DOI: 10.1126/sciadv.abq6974

**The PDF file includes:**

Supplementary Text
Table S1
Legend for movie S1
References

**Other Supplementary Material for this manuscript includes the following:**

Movie S1

# Supplementary Text

In this supplement, we provide a detailed description of the "improved" Kinematic Iceberg Dynamics (iKID) model, which was built upon the existing code base of the original NOAA Geophysical Fluid Dynamics Laboratory (GFDL) KID model (*11, 36*) that was released several years prior. Icebergs are represented as single vertical layer of Lagrangian elements, or particles, joined together by bonds into iceberg "conglomerates". Each element corresponds to a floating column of ice with its own position, velocity, mass, horizontal area, and thickness. Only horizontal forces are represented.

In the original KID model (*11, 36*), each element experiences the following forces: (1) a force due to sea surface slope; (2) a wave radiation force; (3) the Coriolis force, (4) drag from the ocean, atmosphere, and sea ice; and (5) interactive forces between elements. The first three of these forces are identical to those used in point-particle Lagrangian iceberg models (*6,31,37*). The drag forces also take a similar form as given in point-particle iceberg models:

$$\vec{F}_{\mathrm{m}} = \rho_{\mathrm{m}}(0.5c_{\mathrm{m,v}}A_{\mathrm{m,v}} + c_{\mathrm{m,h}}A_{\mathrm{m,h}})|\vec{u}_{\mathrm{m}} - \vec{u}|(\vec{u}_{\mathrm{m}} - \vec{u}) \tag{1}$$

where index m corresponds to the medium of the drag force: ocean (o), air (a), or sea-ice (si). Parameter $\rho_{\mathrm{m}}$ is the density of the medium, and $\vec{u}_{\mathrm{m}}$ and $\vec{u}$ are the velocities of the medium and iceberg particle, respectively. Parameters $c_{\mathrm{m,v}}$ and $c_{\mathrm{m,h}}$ are drag coefficients applied to the vertical (v) and horizontal (h) faces of the particle, which have areas $A_{\mathrm{m,v}}$ and $A_{\mathrm{m,h}}$, respectively. Note that in the bonded-particle model, $A_{\mathrm{m,v}}$ is the area of a vertical particle face that constitutes an edge of the overall bonded-particle iceberg conglomerate, e.g. $A_{\mathrm{m,v}} = 0$ for elements that lie fully within the interior of a conglomerate. The interactive force is a damped elastic force that prevents excessive overlapping between elements, and for bonded elements, also acts against separation so that the elements collectively drift as a rigid body. The interactive force between

two elements, *i* and *j*, is given by

$$(\vec{F}_{\mathrm{IA}})_{ij} = \begin{cases} (\vec{F}_{\mathrm{e}})_{ij} + (\vec{F}_{\mathrm{d}})_{ij} & \text{if } (d_{ij} \leq L_{ij}) \text{ or } (d_{ij} > L_{ij} \text{ and } B_{ij} = 1) \\ 0 & \text{if } d_{ij} > L_{ij} \text{ and } B_{ij} = 0 \ . \end{cases} \tag{2}$$

where $(\vec{F}_{\mathrm{e}})_{ij}$ and $(\vec{F}_{\mathrm{d}})_{ij}$ are the elastic and damped components of the interactive force, $d_{ij}$ is the distance between the elements, and $L_{ij}$ is a critical-interactive-length scale typically set to the sum of the radii of the two elements. Bond matrix $B_{ij} = 1$ if elements *i* and *j* are bonded together and $B_{ij} = 0$ otherwise. The elastic and damped components of the interactive force are given by

$$(\vec{F}_{\mathrm{e}})_{ij} = -\kappa_{\mathrm{e}}(d_{ij} - L_{ij}) m_{ij} \vec{r}_{ij} \tag{3}$$

and

$$(\vec{F}_{\mathrm{d}})_{ij} = -m_{ij} c_{r_{\parallel}} P_{\vec{r}_{ij}} \cdot (\vec{u}_i - \vec{u}_j) \tag{4}$$

respectively. In equations (3) and (4), $m_{ij}$ is the minimum of the masses of the two elements and $\vec{r}_{ij}$ is the directional unit vector between the position of element *i* and *j*. In the first term on the right hand side of equation (3), $\kappa_{\mathrm{e}}$ is the spring constant, $c_{r_{\parallel}}$ is the damping coefficient parallel to $\vec{r}_{ij}$, and $P_{\vec{r}_{ij}}$ is the projection matrix that projects onto $\vec{r}_{ij}$. The interactive forces resemble those used in traditional discrete element methods, but in the original KID formulation, shear forces and bending moments are entirely absent.

For the iKID model, we made three major modifications to the original KID formulation: (1) we developed a multiple time step (MTS) velocity Verlet scheme to efficiently integrate the equations of motion; (2) we added rotational degrees of freedom and modified forces to account for horizontal bending moments and shear forces during bonded-particle interaction, and grounding drag; and (3) we added a stress criterion for breaking bonds to simulate fracture. The

goal in developing an MTS scheme was to increase the maximum stable time step increment of the bonded-particle model ($\Delta t$) from on the order of tens of seconds, to matching the increment used in global circulation models ($\Delta t \geq 30$ min). Such large time steps significantly reduce the computational expense of the overall iceberg module. Our MTS velocity Verlet scheme is based on similar schemes that were developed for molecular dynamics simulations (*38, 39*), where we categorize each force as a "slow" or "fast" component that is evaluated at longer or shorter time step increments, respectively. Here, all forces are evaluated over the long time step increment ($\Delta t$) except grounding drag and interactive forces between elements belonging to the same tabular iceberg conglomerate of bonded elements, which are evaluated over the short time step increment ($\Delta \hat{t}$). The short time step forces are evaluated more frequently using a series of $k$ shorter sub-steps ($\Delta \hat{t} = \Delta t/k$) small enough to guarantee stability. Fracture, or bond breaking, is also determined on the short time step. Denoting the accelerations associated with the "long-step" forces as $\alpha$ and with the "short-step" forces as $\beta$, the iKID-MTS scheme is given in Algorithm 1.

**Algorithm 1** iKID-MTS velocity Verlet

$\vec{u}_0 \Leftarrow \vec{u}_n + \frac{\Delta t}{2}(\vec{\alpha}_n^{\text{exp}} + \vec{\alpha}_n^{\text{imp}})$

$\vec{x}_0 \Leftarrow \vec{x}_n$

**for** $m = 0, k-1$ **do**

    $\vec{u}_{m+\frac{1}{2}} \Leftarrow \vec{u}_m + \frac{\Delta \hat{t}}{2}\vec{\beta}_m^{\text{exp}}$

    $\vec{x}_{m+1} \Leftarrow \vec{x}_m + \vec{u}_m \Delta \hat{t} + \frac{\Delta \hat{t}^2}{2}(\vec{\beta}_m^{\text{exp}})$

    $\vec{u}_{m+1} \Leftarrow \vec{u}_{m+\frac{1}{2}} + \frac{\Delta \hat{t}}{2}\vec{\beta}_{m+1}^{\text{exp}}$

    break bonds wherever $\sigma^{\text{max}} \geq \sigma_{\text{c}}$

**end for**

$\vec{x}_{n+1} \Leftarrow \vec{x}_k$ †

**if** contact between conglomerates **then**

    $\vec{u}^* \Leftarrow \vec{u}_k$

    $\delta \Leftarrow \infty$

    **while** $\delta > \varepsilon$ **do**

        $\vec{u}_{n+1} \Leftarrow \vec{u}^* + \frac{\Delta t}{2}(\vec{\alpha}_k^{\text{exp}} + \vec{\alpha}_{n+1}^{\text{imp}})$

        $\delta \Leftarrow \frac{2\|\vec{u}_{n+1} - \vec{u}^*\|}{\|\vec{u}_{n+1}\| + \|\vec{u}^*\|}$

        **if** $\delta > \varepsilon$ **then**

            $\vec{u}^* \Leftarrow \vec{u}_{n+1}$

        **end if**

    **end while**

**else**

    $\vec{u}_{n+1} \Leftarrow \vec{u}_k + \frac{\Delta t}{2}(\vec{\alpha}_k^{\text{exp}} + \vec{\alpha}_{n+1}^{\text{imp}})$

**end if**

† After this position update, several routines external to the MTS scheme are run, including particle/grid interpolations, parallel transfers, and iceberg melting (though no melting was allowed in the A68a simulation). These processes are not included in the estimates of the MTS clock-time in the Materials and Methods section of the main text. The next computational cycles for any coupled models, e.g. ocean and sea ice, are also run at this point.

**iKID-MTS: Long time steps**

In Algorithm 1, the short time steps are represented by the loop on line 3, while the rest of the algorithm is evaluated on the long time step. Note that all long-step velocity updates require calculating both explicit ($\alpha^{\text{exp}}$) and implicit accelerations ($\alpha^{\text{imp}}$). The ocean, wind, and sea-ice drag terms are evaluated implicitly, i.e. using the velocity at the current time step. The damping term for contact between elements that belong to different conglomerates, which is also evaluated on the long time step, is implicit as well. For this damping term (4), we set $c_{r_{\|}} = 2\sqrt{\kappa_{\text{e}}}$ so that the contact force is critically damped parallel to $\vec{r}_{ij}$. The Coriolis term is integrated using a semi-implicit Crank-Nicolson scheme. We refer the reader to the description of the original KID model (*36*) for an in-depth discussion on how these acceleration terms are calculated, which involves using a predictive-corrective scheme for the drag terms. Here, calculation of long-step accelerations is the same as in the original KID scheme with two exceptions: (1) interactive forces between elements belonging to the same conglomerate are instead evaluated on the short-step, and (2) some additional treatment of long-step contact forces, as explained in the remainder of this section.

Given that interactive forces are rapid processes, it may seem counter-intuitive to evaluate contact between elements that belong to different conglomerates on the long time step. However, this approach allows efficient parallelization by reducing transfers of memory between processors to just once per computational cycle (after line 9 of Algorithm 1). Before the transfers, all grid quantities needed to evaluate the external force balance, i.e. drag velocities, sea surface gradients, and seafloor depth (see *Grounding*), are interpolated to the iceberg elements. Then, when a conglomerate overlaps multiple processing domains, the entire conglomerate and any elements within contact range of the conglomerate are copied to all processors associated with the overlap. Because these transfers occur just once per computational cycle, contact forces between different conglomerates, or with particles without any bonds, must be evaluated during

the long step. If these contact forces were instead evaluated during the short-step loop, repeated transfers between processors each short-step would be needed to account for the repeated element position updates, which may introduce new contact pairs across processors. Such repeated transfers would be computationally-expensive.

Special treatment may be required to evaluate contact between conglomerates as a long time step force, where the goal is to simply prevent overlapping of conglomerates. We must set the long-step spring constant, $\kappa_{\mathrm{e}}$, to a low value so that iceberg conglomerates do not unrealistically "bounce" off of one another upon contact and rapidly accelerate in opposite directions. In turn, this weak long-step spring constant could result in the non-physical overlapping of elements that belong to different conglomerates, which increases in severity at higher particle resolutions (smaller radii). However, this overlapping may be mitigated by increasing the critical-interactive-length scale, $L_{ij}$, i.e. increasing the distance between particles at which the collision force is activated.

Finally, we address the "if" statement on line 10 of the algorithm, which is also related to evaluating inter-conglomerate contact on the long time step. This contact force includes a implicit damping term (4) that depends on the relative velocity of the contacting particles at the current time step. Therefore, in the case of contact between different conglomerates, the while loop on line 10 of the algorithm is needed to iteratively update these velocities to convergence, where $\delta$ is the convergence criteria based on the norm of the change in the velocity solution, and $\varepsilon$ is the convergence tolerance. This iterative scheme is necessary to conserve momentum in cases where such contact does occur, and ensures a stable and consistent fracture response.

**KID-MTS: Short time steps**

During the inner short-step loop, we calculate the interactive forces explicitly according to a scale-invariant bonded-particle model for continua (*20*), which we implement exactly as given

except that we must scale the published non-damping force and torque terms by the bond thickness (here, all bonds and elements are assigned a constant thickness of $H = 200$ m for A68a), because the published equations (*20*) assumed $H = 1$. This fully-explicit short-step model includes horizontal shear forces and bending moments, which helps guarantee a more accurate fracture response and avoids unrealistic spurious bending of "strings" of bonded elements that are only one element in width, but multiple elements in length. Note that the interactive forces evaluated during the short time steps include interaction between both bonded and unbonded element pairs within the same conglomerate. Unbonded elements within the same conglomerate can interact, for example, due to contact between elements on the opposite flanks of a rift. For simplicity, we do not account for shear forces during contact of unbonded elements.

***Grounding***

Grounding is enforced using a linear drag force

$$\vec{F}_{\mathrm{G}} = c_{\mathrm{g}} A \vec{u} \ , \tag{5}$$

where $A$ is the horizontal area of the iceberg element and $c_{\mathrm{g}}$ is a grounding coefficient. We evaluate this force during the short time step to account for the rapid changes in iceberg velocity that can occur when an element first becomes grounded. By default, this grounding drag is evaluated for particles with a keel depth that meets or exceeds the seafloor depth, which is interpolated to iceberg elements using a quadratic interpolation scheme during the long-step. However, for the A68a experiment, grounding drag was only evaluated for particles that overlapped a manually designated grounding zone during the long-step (see Results).

***Fracture***

At the end of each short-step, we break a bond whenever the maximum tensile stress, $\sigma^{\max}$,

meets or exceeds a specified tensile strength, $\sigma_c$. The maximum tensile stress is calculated from beam theory (*21*) as

$$\sigma^{\max} = -\frac{F_n}{A_b} + \frac{|M_r| W_b}{2I} \tag{6}$$

where $F_n$ is the normal force carried by the bond, $M_r$ is the bending moment caused by the relative rotation of the two bonded particles, $W_b$ is the bond width, $A_b = W_b H$ is the area of the bond cross-section, and $I = \frac{W_b^3 H}{12}$ is the moment of inertia of the bond. Note that for the A68a experiments, all elements have the same radius, $R = 1.5$ km, so that the bond width $W_b = 2R = 3$ km.

Bonds may also be broken whenever the maximum shear stress, $\tau^{\max}$ meets or exceeds a specified shear strength, $\tau_c$. The maximum shear stress is calculated as

$$\tau^{\max} = -\frac{|F_s|}{A_b} \tag{7}$$

where $F_s$ is the shearing force carried by the bond. Because iceberg A68a appears to fracture under tension, we neglect the shear stress bond-breakage criterion here.

Note that implementing the fracture criterion affects how we tune the size of the short time step increment, $\Delta\hat{t}$. This increment is typically set so that perturbations to an element do not propagate further than its immediate neighbors over a single time step, where larger element sizes and smaller stiffnesses allow for larger time step increments (*40*). Here, we also tune $\Delta\hat{t}$ so that the fracture response does not significantly differ if an even smaller $\Delta\hat{t}$ is chosen.

***Melting***

While we set melt rates to zero for the A68a simulation, the KID-MTS framework uses the same melt model as the original KID formulation (*36*), which is based on a combination of melting parameterizations that are typically used in iceberg drift (*6*) and ice shelf (*41*) mod-

els. As an element melts, its tracked mass and horizontal dimensions is decreased accordingly. Note, however, that unlike in the original KID formulation, these tracked quantities are not used in the MTS model when calculating forces for elements with bonds. Instead, forces for these elements are calculated using their original radius and horizontal areas, as well as an artificial mass calculated from the product of their thickness, density, and original horizontal area. Without these modifications, the maximum stable $\Delta\hat{t}$ for sub-step element-interaction forces would decrease as element size decreases.

**Table S1:** Iceberg A68a experiment parameters

| Parameter | Description | Value |
|---|---|---|
| $H$ | Ice thickness | 200 m |
| $\rho$ | Ice density | 850 kg/m$^3$ |
| $\Delta t$ | Long-step time increment | 0.5 hr |
| $\kappa_{\mathrm{e}}$ | Spring constant (long-step) | $10^{-7}$ s$^{-2}$ |
| $c_{r_{\|}}$ | Damping coefficient (long-step) | $2\sqrt{\kappa_e}$ |
| $L_{ij}$ | Critical-interactive-length scale (long-step) | 4 km |
| $\varepsilon$ | Convergence tolerance for long-steps with contact | $10^{-4}$ |
| $\Delta\hat{t}$ | Short-step time increment | 20 s |
| $k$ | Number of short-steps per long-step | 90 |
| $E$ | Young's Modulus (short-step)[1] | 5 MPa |
| $\nu$ | Poisson's Ratio (short-step)[1] | 0.3 |
| $C$ | Damping coefficient (short-step)[1] | 1 |
| $\sigma_{\mathrm{c}}$ | Tensile bond strength | 18 kPa |
| $c_{\mathrm{g}}$ | Grounding coefficient | $10^4$ kg m$^{-2}$ s$^{-1}$ |
| $c_{\mathrm{o,h}}$ | Horizontal ocean drag coefficient | 0.02136 |
| $c_{\mathrm{o,v}}$ | Vertical ocean drag coefficient | 16.02 |
| $c_{\mathrm{a,h}}$ | Horizontal wind drag coefficient | 0.0055 |
| $c_{\mathrm{a,v}}$ | Vertical wind drag coefficient | 1.3 |

[1]These parameters are part of the explicit bonded-particle model (*20*) used to evaluate the short-step interactive forces.

**Movie S1** The simulated drift and decay of iceberg A68a in December, 2020. This is the animation corresponding to Figure 2. The first breakup event occurs when the iceberg contacts the seafloor around Dec 17th, where the modeled grounding zone is represented by the orange box. The second breakup event, around Dec 20th, is caused by strong shear in ocean currents (arrows).

## REFERENCES AND NOTES


1. M. A. Depoorter, J. L. Bamber, J. A. Griggs, J. T. M. Lenaerts, S. R. M. Ligtenberg, M. R. van den Broeke, G. Moholdt, Calving fluxes and basal melt rates of Antarctic ice shelves. *Nature* **502**, 89–92 (2013).

2. E. Rignot, J. Mouginot, B. Scheuchl, Ice-shelf melting around Antarctica. *Science* **341**, 266–270 (2013).

3. J. Tournadre, N. Bouhier, F. Girard-Ardhuin, F. Rémy, Antarctic icebergs distributions 1992–2014. *J. Geophys. Res. Oceans* **121**, 327–349 (2016).

4. J. S. Budge, D. G. Long, A comprehensive database for Antarctic iceberg tracking using scatterometer data. *IEEE J. Sel. Top. Appl. Earth Obs. Remote Sens.* **11**, 434–442 (2018).

5. J. I. Jongma, E. Driesschaert, T. Fichefet, H. Goosse, H. Renssen, The effect of dynamic–thermodynamic icebergs on the Southern Ocean climate in a three-dimensional model. *Ocean Model.* **26**, 104–113 (2009).

6. T. Martin, A. Adcroft, Parameterizing the fresh-water flux from land ice to ocean with interactive icebergs in a coupled climate model. *Ocean Model.* **34**, 111–124 (2010).

7. R. Marsh, V. O. Ivchenko, N. Skliris, S. Alderson, G. R. Bigg, G. Madec, A. T. Blaker, Y. Aksenov, B. Sinha, A. C. Coward, J. le Sommer, N. Merino, V. B. Zalesny, NEMO–ICB (v1.0): Interactive icebergs in the NEMO ocean model globally configured at eddy-permitting resolution. *Geosci. Model Dev.* **8**, 1547–1562 (2015).

8. A. Starr, I. R. Hall, S. Barker, T. Rackow, X. Zhang, S. R. Hemming, H. J. L. van der Lubbe, G. Knorr, M. A. Berke, G. R. Bigg, A. Cartagena-Sierra, F. J. Jiménez-Espejo, X. Gong, J. Gruetzner, N. Lathika, L. J. Le Vay, R. S. Robinson, M. Ziegler; Expedition 361 Science Party, Antarctic icebergs reorganize ocean circulation during Pleistocene glacials. *Nature* **589**, 236–241 (2021).

9. H. Heinrich, Origin and consequences of cyclic ice rafting in the Northeast Atlantic Ocean during the past 130,000 years. *Quatern. Res.* **29**, 142–152 (1988).

10. A. Condron, J. C. Hill, Timing of iceberg scours and massive ice-rafting events in the subtropical North Atlantic. *Nat. Commun.* **12**, 3668 (2021).

11. A. A. Stern, A. Adcroft, O. Sergienko, Modeling ice shelf cavities and tabular icebergs using Lagrangian elements. *J. Geophys. Res. Oceans* **124**, 3378–3392 (2019).

12. A. Huth, A. Adcroft, O. Sergienko, Parameterizing tabular-iceberg decay in an ocean model. *J. Adv. Model. Earth Syst.* **14**, e2021MS002869 (2022).

13. M. R. England, T. J. W. Wagner, I. Eisenman, Modeling the breakup of tabular icebergs. *Sci. Adv.* **6**, eabd1273 (2020).

14. A. Stern, A. Adcroft, O. Sergienko, The effects of Antarctic iceberg calving-size distribution in a global climate model. *J. Geophys. Res. Oceans* **121**, 5773–5788 (2016).

15. E. W. Hester, C. D. McConnochie, C. Cenedese, L.-A. Couston, G. Vasil, Aspect ratio affects iceberg melting. *Phys. Rev. Fluids* **6**, 023802 (2021).

16. A. Braakmann-Folgmann, A. Shepherd, L. Gerrish, J. Izzard, A. Ridout, Observing the disintegration of the A68A iceberg from space. *Remote Sens. Environ.* **270**, 112855 (2022).

17. F. Parmiggiani, M. Moctezuma-Flores, L. Guerrieri, M. L. Battagliere, SAR analysis of the Larsen-C A-68 iceberg displacements. *Int. J. Remote Sens.* **39**, 5850–5858 (2018).

18. T. Scambos, R. Ross, R. Bauer, Y. Yermolin, P. Skvarca, D. Long, J. Bohlander, T. Haran , Calving and ice-shelf break-up processes investigated by proxy: Antarctic tabular iceberg evolution during northward drift. *J. Glaciol.* **54**, 579–591 (2008).

19. T. Scambos, O. Sergienko, A. Sargent, D. MacAyeal, J. Fastook, ICESat profiles of tabular iceberg margins and iceberg breakup at lowlatitudes. *Geophys. Res. Lett.* **32**, L23S09 (2005).

20. M. Wang, A scale-invariant bonded particle model for simulating large deformation and failure of continua. *Comput. Geotech.* **126**, 103735 (2020).

21. D. O. Potyondy, P. A. Cundall, *Int. J. Rock Mech. Min. Sci.* **41**, A bonded-particle model for rock. 1329–1364 (2004).

22. ESR, Oscar third deg (2009).

23. SSALTO/DUACS, Near-real-time absolute dynamic topography (2021).

24. E. Kalnay, M. Kanamitsu, R. Kistler, W. Collins, D. Deaven, L. Gandin, M. Iredell, S. Saha, G. White, J. Woollen, Y. Zhu, A. Leetmaa, R. Reynolds, M. Chelliah, W. Ebisuzaki, W. Higgins, J. Janowiak, K. C. Mo, C. Ropelewski, J. Wang, R. Jenne, D. Joseph, The NCEP/NCAR 40-year reanalysis project. *Bull. Am. Meteorol. Soc.* **77**, 437–471 (1996).

25. N. K. Sinha, Short-term rheology of polycrystalline ice. *J. Glaciol.* **21**, 457–474 (1978).

26. P. Duval, In Canaan. *IAHS Publ.* **118**, 29 (1977).

27. L. Lliboutry, P. Duval, Various isotropic and anisotropic ices found in glaciers and polar ice caps and their corresponding rheologies. *Int. J. Rock Mech. Min. Sci. Geomech. Abstr.* **22**, 198 (1985).

28. C. Mosbeux, T. J. W. Wagner, M. K. Becker, H. A. Fricker, Viscous and elastic buoyancy stresses as drivers of ice-shelf calving. *J. Glaciol.* **66**, 643–657 (2020).

29. D. McGrath, K. Steffen, T. Scambos, H. Rajaram, G. Casassa, J. L. Rodriguez Lagos, Basal crevasses and associated surface crevassing on the Larsen C ice shelf, Antarctica, and their role in ice-shelf instability. *Ann. Glaciol.* **53**, 10–18 (2012).

30. A. Luckman, D. Jansen, B. Kulessa, E. C. King, P. Sammonds, D. I. Benn, Basal crevasses in Larsen C Ice Shelf and implications for their global abundance. *Cryosphere* **6**, 113–123 (2012).

31. R. M. Gladstone, G. R. Bigg, K. W. Nicholls, Iceberg trajectory modeling and meltwater injection in the Southern Ocean. *J. Geophys. Res. Oceans* **106**, 19903–19915 (2001).

32. T. J. W. Wagner, R. W. Dell, I. Eisenman, An analytical model of iceberg drift. *J. Phys. Oceanogr.* **47**, 1605–1616 (2017).

33. D. R. MacAyeal, M. H. Okal, J. E. Thom, K. M. Brunt, Y. J. Kim, A. K. Bliss, Tabular iceberg collisions within the coastal regime. *Journal of Glaciology* **54**, 371–386 (2008).

34. GEBCO Compilation Group, Gebco 2021 grid (2021).

35. O. T. Hogg, V. A. I. Huvenne, H. J. Griffiths, B. Dorschel, K. Linse, Landscape mapping at sub-Antarctic South Georgia provides a protocol for underpinning large-scale marine protected areas. *Sci. Rep.* **6**, 33163 (2016).

36. A. A. Stern, A. Adcroft, O. Sergienko, G. Marques, Modeling tabular icebergs submerged in the ocean. *J. Adv. Model. Earth Syst.* **9**, 1948–1972 (2017).

37. G. R. Bigg, M. R. Wadley, D. P. Stevens, J. A. Johnson, Modelling the dynamics and thermodynamics of icebergs. *Cold Reg. Sci. Technol.* **26**, 113–135 (1997).

38. M. Tuckerman, B. J. Berne, G. J. Martyna, Reversible multiple time scale molecular dynamics. *J. Chem. Phys.* **97**, 1990–2001 (1992).

39. H. Grubmüller, H. Heller, A. Windemuth, K. Schulten, Generalized Verlet algorithm for efficient molecular dynamics simulations with long-range interactions. *Mol. Simul.* **6**, 121–142 (1991).

40. M. Wang, Y. Feng, C. Wang, Coupled bonded particle and lattice Boltzmann method for modelling fluid-solid interaction. *Int. J. Numer. Anal. Methods Geomech.* **40**, 1383–1401 (2016).

41. D. M. Holland, A. Jenkins, Modeling thermodynamic ice–ocean interactions at the base of an ice shelf. *J. Phys. Oceanogr.* **29**, 1787–1800 (1999).
